# LncRNA *RPPH1* promotes colorectal cancer metastasis by interacting with TUBB3 and by promoting exosomes-mediated macrophage M2 polarization

**DOI:** 10.1038/s41419-019-2077-0

**Published:** 2019-11-04

**Authors:** Zhen-xing Liang, Hua-shan Liu, Feng-wei Wang, Li Xiong, Chi Zhou, Tuo Hu, Xiao-wen He, Xiao-jian Wu, Dan Xie, Xian-rui Wu, Ping Lan

**Affiliations:** 1 Department of Colorectal Surgery, The Sixth Affiliated Hospital, Sun Yat-sen University, Guangzhou, Guangdong China; 2 Guangdong Provincial Key Laboratory of Colorectal and Pelvic Floor Diseases, The Sixth Affiliated Hospital, Sun Yat-sen University, Guangzhou, Guangdong China; 3 Guangzhou Regenerative Medicine and Health Guangdong Laboratory, Guangzhou, China; 4 State Key Laboratory of Oncology in South China, Collaborative Innovation Center for Cancer Medicine, Sun Yat-sen University Cancer Center, Guangzhou, Guangdong China; 5 Department of Endocrinology, The First Affiliated Hospital of Sun Yat-sen University, Guangzhou, China

**Keywords:** Cancer microenvironment, Colorectal cancer

## Abstract

Metastasis is a well-known poor prognostic factor in cancer. However, the mechanisms how long non-coding RNAs (lncRNAs) regulate metastasis in colorectal cancer (CRC) remain largely unknown. Besides, tumor-associated macrophages (TAMs) play an important role in tumor progression, yet the contribution of lncRNA-mediated crosstalk between TAMs and CRC cells to tumor progression is not well understood. In this study, we report that lncRNA *RPPH1* was significantly upregulated in CRC tissues, and the *RPPH1* overexpression was associated with advanced TNM stages and poor prognosis. *RPPH1* was found to promote CRC metastasis in vitro and in vivo. Mechanistically, *RPPH1* induced epithelial–mesenchymal transition (EMT) of CRC cells via interacting with β-III tubulin (TUBB3) to prevent its ubiquitination. Furthermore, CRC cell-derived exosomes transported *RPPH1* into macrophages which mediate macrophage M2 polarization, thereby in turn promoting metastasis and proliferation of CRC cells. In addition, exosomal *RPPH1* levels in blood plasma turned out to be higher in treatment-naive CRC patients but lower after tumor resection. Compared to CEA and CA199, exosomal *RPPH1* in CRC plasma displayed a better diagnostic value (AUC = 0.86). Collectively, *RPPH1* serves as a potential therapeutic and diagnostic target in CRC.

## Introduction

Colorectal cancer (CRC) is one of the most prevalent cancers in the world^1^. It accounted for 8% of cancer incidence and 8% of the cancer-related deaths in 2018^2^. The poor prognosis is largely due to the distant metastasis, which accounts for about 90% of cancer-related deaths^3,4^. Surgical resection with or without chemo-/radiotherapy is the standard treatment of the primary tumor, but therapeutic options for distant metastasis are limited. Metastasis is a complex process, and the molecular mechanisms remain largely unknown. Therefore, it is urgently necessary to explore the molecular mechanisms and promote the development of therapy targeting metastasis.

Long non-coding RNAs (lncRNAs) are a large class of transcripts longer than 200 bp with a limited protein-coding potential^5^. Many studies have shown that lncRNAs participate in a series of biological processes, including epigenetic, transcriptional, and post-transcriptional levels, as well as the initiation and progression of cancers^6–9^. Emerging evidence suggests that lncRNAs participate in every stage of metastasis from cell migration to distant-organ colonization^10^. For example, colon cancer associated transcript 2 (*CCAT2*) is upregulated in CRC and can promote cancer cell migration and metastasis^11^. LncRNA-activated by TGF-β (lncRNA-ATB) can bind to the miR-200 family and IL-11 mRNA to promote the invasion-metastasis cascade^12^. Although many lncRNAs have been reported to be involved in tumor biological processes, the mechanisms linking metastasis and lncRNAs are still largely unknown.

Exosomes are microvesicles ranging from 70 to 120 nm in diameter and are derived from multivesicular bodies^13^. Exosomes participate in cells communication by transferring proteins and nucleic acids^14,15^. In recent studies, many exosomal proteins, miRNAs, and lncRNAs were reported to promote tumor progression^16–19^. For example, lncARSR can be transported by exosomes from sunitinib-resistant cells to sensitive cells, thereby promoting sunitinib resistance in renal cell carcinoma^18^. However, the relationships between exosomal lncRNAs and metastasis in CRC need to be further studied.

Tumor-associated macrophages (TAMs) are the most abundant cells in tumor microenvironment^20,21^. Numerous studies show that TAMs are associated with poor prognosis and may promote tumor progression and metastasis^22,23^. TAMs may become a therapeutic target in the future. But why macrophages polarize to TAMs in tumor microenvironment is puzzling.

In the present study, we found that *RPPH1* is upregulated in CRC specimens and associated with advanced TNM stage and poor prognosis. *RPPH1* was found to promote CRC cells migration and invasion in vitro and in vivo. Mechanistically, *RPPH1* binds to β-III tubulin (TUBB3) to prevent its ubiquitination and then induces epithelial–mesenchymal transition (EMT) to promote metastasis. Furthermore, we found that *RPPH1* can be transferred by exosomes to macrophages to mediate macrophage M2 polarization. Exosomal *RPPH1* turned out to be upregulated in the blood plasma of CRC patients, but downregulated afterwards. These findings indicate that *RPPH1* may be a potential diagnostic marker and therapeutic target in CRC.

## Results

### Upregulated *RPPH1* is associated with poor prognosis in CRC patients

**Fig. 1:**
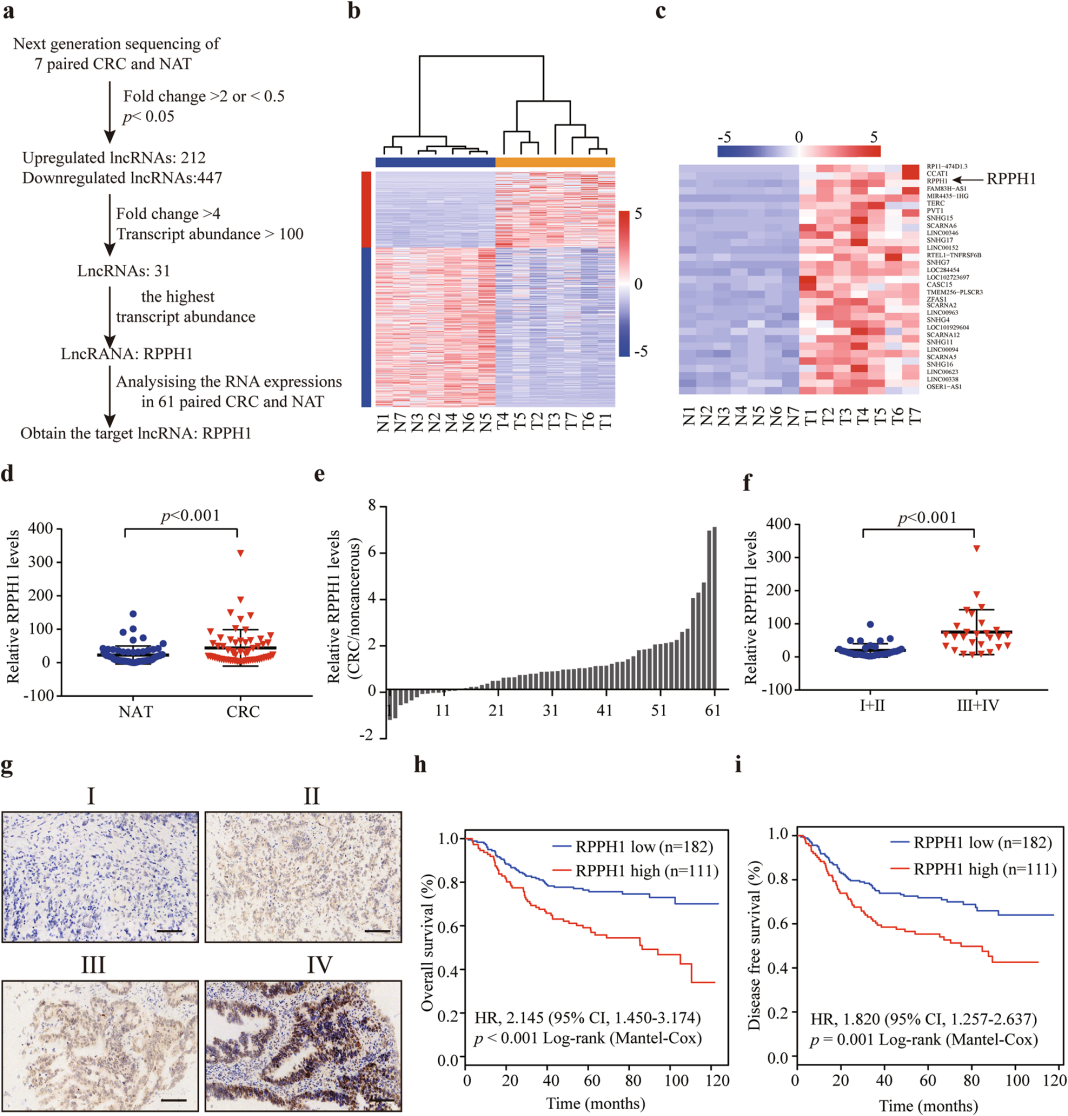
*RPPH1* is associated with poor prognosis in CRC patients. **a** The schematic of screening target lncRNAs. **b** Heatmap of the screening lncRNAs with a fold change > 2 or <0.5 and *p* < 0.05 from seven CRC tissues and paired normal adjacent tissues (NATs). **c** Heatmap of 31 lncRNAs with a fold change > 4 and transcript abundance >100. **d** *RPPH1* RNA levels were quantified in 61 paired of CRC tissues and NATs using qRT-PCR. **e** Fold change of *RPPH1* expression levels in 61 paired CRC tissues. **f** The relationship between *RPPH1* expression levels and TNM stage. **g** Representative images of the ISH of RPPH1 expression (brown) in the paraffin-embedded CRC tissues of different TNM stages. Scale bar = 100 μm. **h**, **i** Kaplan–Meier curves for OS (**h**) and DFS (**i**) of CRC patients with low vs. high expression of RPPH1. Values are represented as mean ± SD

To identify lncRNAs which contribute to metastasis, next-generation sequencing (NGS) was performed on paired CRC samples and normal adjacent tissues (NATs) from seven CRC patients with liver metastasis. These seven patients’ detailed characteristics are shown in Supplementary Table 1. The flow chart of screening aimed at finding the target lncRNAs is presented in Fig. 1a. There were 212 upregulated and 447 downregulated lncRNAs with fold change > 2 or <0.5 and *p* < 0.05 (Fig. 1b). To identify the target lncRNAs, 31 lncRNAs were reserved with more rigorous criteria, fold change > 4, and transcript abundance >100 (Fig. 1c). Then, we chose the lncRNA with highest transcript abundance, lncRNA *RPPH1*, as the final target. *RPPH1*, Ribonuclease P RNA component H1, is the RNA component of the RNase P ribonucleoprotein^24^. Recent studies revealed that *RPPH1* is upregulated in gastric cancer and breast cancer, but the mechanisms are not yet clear^25,26^. We next quantified *RPPH1* in 61 paired CRC samples and NATs by quantitative real-time PCR (qRT-PCR) analysis (*p* < 0.001, Fig. 1d). *RPPH1* expression was found to be upregulated in 78.7% (48/61) of CRC patients (Fig. 1e). Statistical analysis revealed that *RPPH1* levels were strongly associated with advanced TNM stages (III and IV; *p* < 0.001, Fig. 1f). *RPPH1* locates in chromosome 14q11.2 and the 5′ and 3′ rapid amplification of cDNA ends (RACE) assays were performed to characterize the full-length *RPPH1* in CRC cells (Supplementary Fig. 1a–c). Then the coding potential of *RPPH1* was analyzed by the Coding Potential Assessment Tool (CPAT), the Coding Potential Calculator (CPC), and PhyloCSF codon substitution frequency analysis (Supplementary Fig. 1d–f). All these analyses showed that *RPPH1* is a non-coding RNA.

Furthermore, we analyzed *RPPH1* expression in a large cohort of CRC patients by in situ hybridization (ISH; *n* = 293, Supplementary Table 2). The ISH analysis also suggested that *RPPH1* expression was higher in advanced TNM stages (Fig. 1g) and was associated with metastasis (*p* < 0.001, Supplementary Table 2). An optimized cutoff of *RPPH1* expression was generated using R (maxstat package) for the following analysis. Survival analysis showed that high *RPPH1* expression was associated with poor overall survival (OS; *p* < 0.001) and disease-free survival (DFS) in CRC patients (*p* = 0.001; Fig. 1h, i). Taken together, these data suggested that *RPPH1* plays an important role in CRC progression.

### *RPPH1* promotes CRC cell migration, invasion, and EMT in vitro

**Fig. 2:**
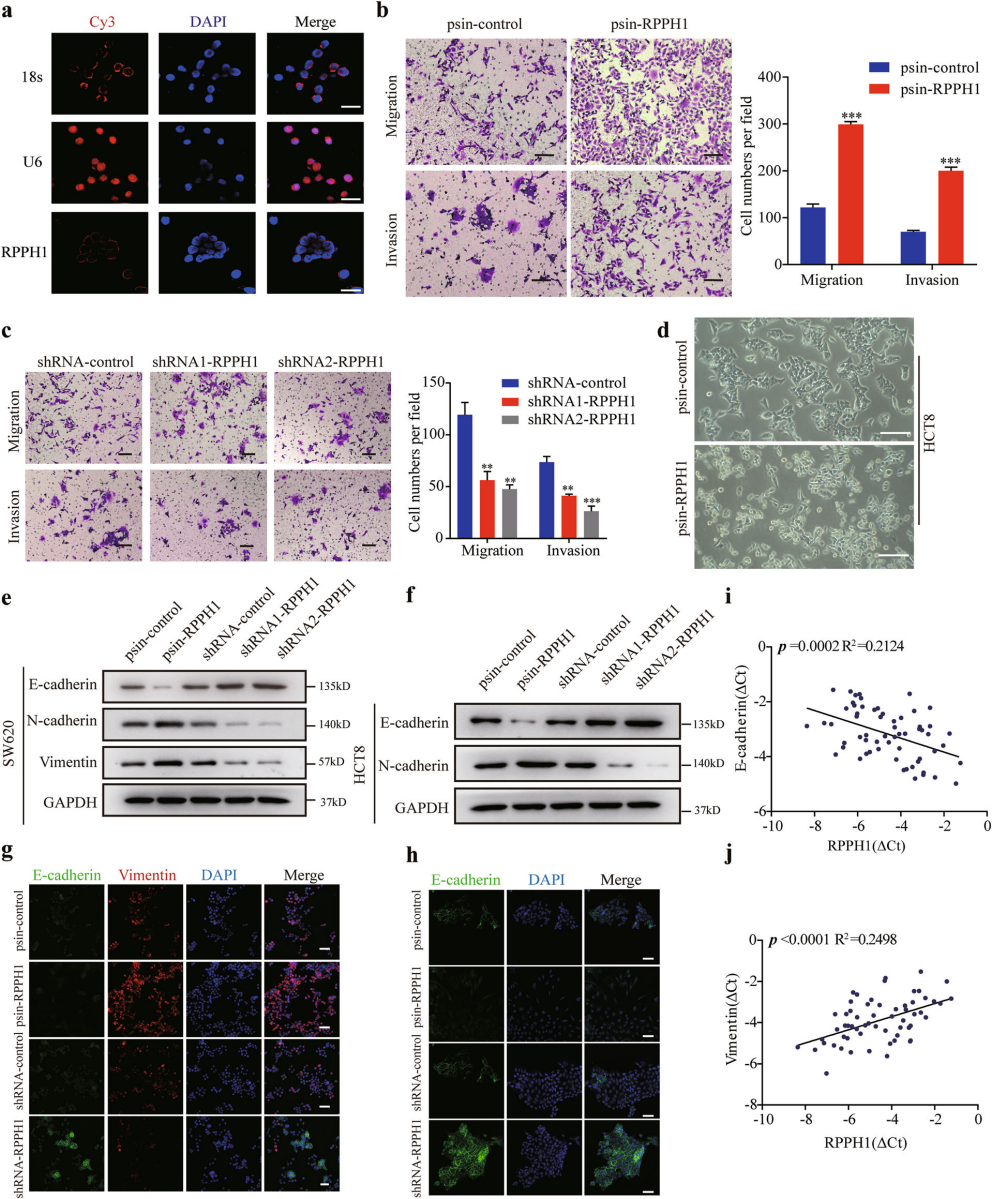
*RPPH1* increases CRC cells migration, invasion, and EMT in vitro. **a** FISH analysis of the subcellular distribution of RPPH1 in HT29 cells. Scale bar = 20 μm. **b**, **c** Transwell migration and invasion assays in stable RPPH1 overexpression (**b**) or knockdown (**c**) HCT8 cells. Scale bar = 100 μm. **d** Representative images of HCT8 cells and RPPH1-overexpressing HCT8 cells. Scale bar = 40 μm. **e**, **f** The EMT effect was validated by WB analysis of epithelial or mesenchymal markers in SW620 (**e**) and HCT8 cells (**f**). **g**, **h** The EMT effect was further confirmed by immunofluorescence analysis in SW620 (**g**) and HCT8 cells (**h**). Scale bar = 40 μm. **i**, **j** The correlation between RPPH1 expression level and E-cadherin (**i**) or Vimentin (**j**) mRNA level was measured in 61 paired CRC tissues. The ∆Ct values (normalized to GUSB) were subjected to Pearson correlation analysis. Values are represented as mean ± SD. **p < 0.01 and ***p < 0.001

To investigate the functions of *RPPH1* in CRC, we first analyzed the relative expression levels of *RPPH1* in CRC cell lines (Supplementary Fig. 2a) and HCT8 and SW620 cells were chosen to perform the following assays because of their moderate *RPPH1* expression levels compared to other CRC cell lines. Then we performed fluorescence in situ hybridization (FISH) to identify the location of *RPPH1*. As shown in Fig. 2a and Supplementary Fig. 2b, *RPPH1* mainly localized in the cytoplasm. We constructed stable *RPPH1* overexpression and knockdown cells in SW620 and HCT8 cells and quantified the *RPPH1* levels by qRT-PCR (Supplementary Fig. 2c, d). *RPPH1* overexpression obviously enhanced the migration and invasion abilities of HCT8 (Fig. 2b) and SW620 cells (Supplementary Fig. 2e). On the contrary, the *RPPH1* knockdown decreased the migration and invasion abilities of the cells as assessed by migration and invasion assays (Fig. 2c, Supplementary Fig. 2f). However, the changes of *RPPH1* expression had no impact on cells proliferation abilities according to the MTS assay (Supplementary Fig. 2g). While we cultured *RPPH1*-overexpressing HCT8 cells, we accidentally found these cells acquired mesenchyma-like morphological features (Fig. 2d). Hence, we next performed western blots and immunofluorescence assay to analyze the changes of EMT markers in CRC cells. We found *RPPH1* overexpression increased the expression of mesenchymal markers N-cadherin and vimentin but reduced epithelial markers E-cadherin in SW620 and HCT8 cells (Fig. 2e–h). Likewise, the *RPPH1* knockdown cells upregulated E-cadherin, but downregulated N-cadherin and vimentin (Fig. 2e–h). Furthermore, we analyzed the mRNA levels of EMT markers in CRC tissues to confirm the correlation between *RPPH1* and EMT. As shown in Fig. 2i, j, we found that the *RPPH1* transcript level was correlated with the vimentin mRNA level, but negatively correlated with the E-cadherin mRNA level. Altogether, those results suggested that *RPPH1* may play a vital role in CRC cells metastasis.

### *RPPH1* promotes CRC cell metastasis in vivo

**Fig. 3:**
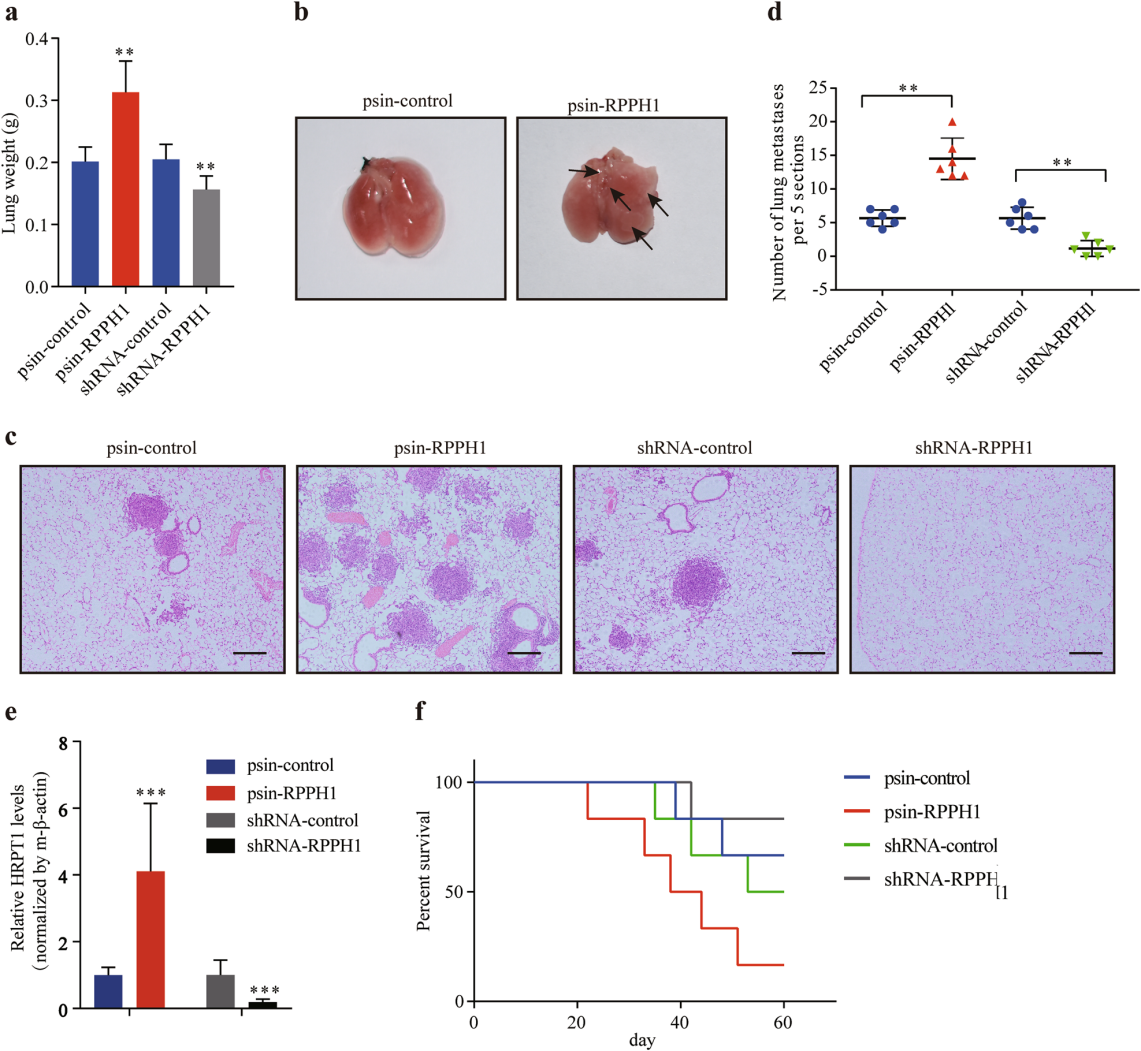
*RPPH1* promotes CRC cells metastasis in vivo. **a** Wet lung weight in nude mice with SW620 cells injected via tail vein. **b** Autopsy of the lungs in the nude mice. **c** H&E of the lungs in the nude mice. Scale bar = 100 μm. **d** Number of metastatic nodules in the lungs from the nude mice via H&E (five sections evaluated per lung). **e** Expression of human HPRT1 mRNA levels relative to mouse β-actin in the lungs. **f** Kaplan–Meier survival curve for the nude mice. *n* = 6 per group. Values are represented as mean ± SD. **p *<* 0.01 and ***p *<* 0.001

It is well known that EMT is a crucial step in the early stages of tumor cells metastasis^27^ and we found that *RPPH1* can promote EMT in CRC cells. Therefore, we further investigated whether *RPPH1* can promote CRC cells metastasis in vivo. The SW620 cells with stable overexpression of *RPPH1* or hairpin RNA (shRNA) targeting *RPPH1* were injected into nude mice via tail vein (*n* = 6 per group). Eight weeks later, all the mice were euthanized, and we analyzed the lung metastasis. We found the lungs of the *RPPH1* overexpression group were heavier than those in the control group, and the lungs in the *RPPH1* knockdown group were lighter (Fig. 3a). Autopsy and hematoxylin and eosin staining (H&E) analysis also showed *RPPH1* overexpression led to more metastasis in the lungs (Fig. 3b–d). Quantification of human HPRT mRNA levels in mouse lungs also suggested that *RPPH1* promoted lung metastasis of CRC cells (Fig. 3e). Moreover, Kaplan–Meier analysis revealed that *RPPH1* overexpression reduced the survival of mice, whereas the *RPPH1* knockdown had the opposite effect (Fig. 3e). Collectively, these data meant that *RPPH1* enhanced the metastasis ability of CRC.

### *RPPH1* physically interacts with TUBB3 in CRC cells

**Fig. 4:**
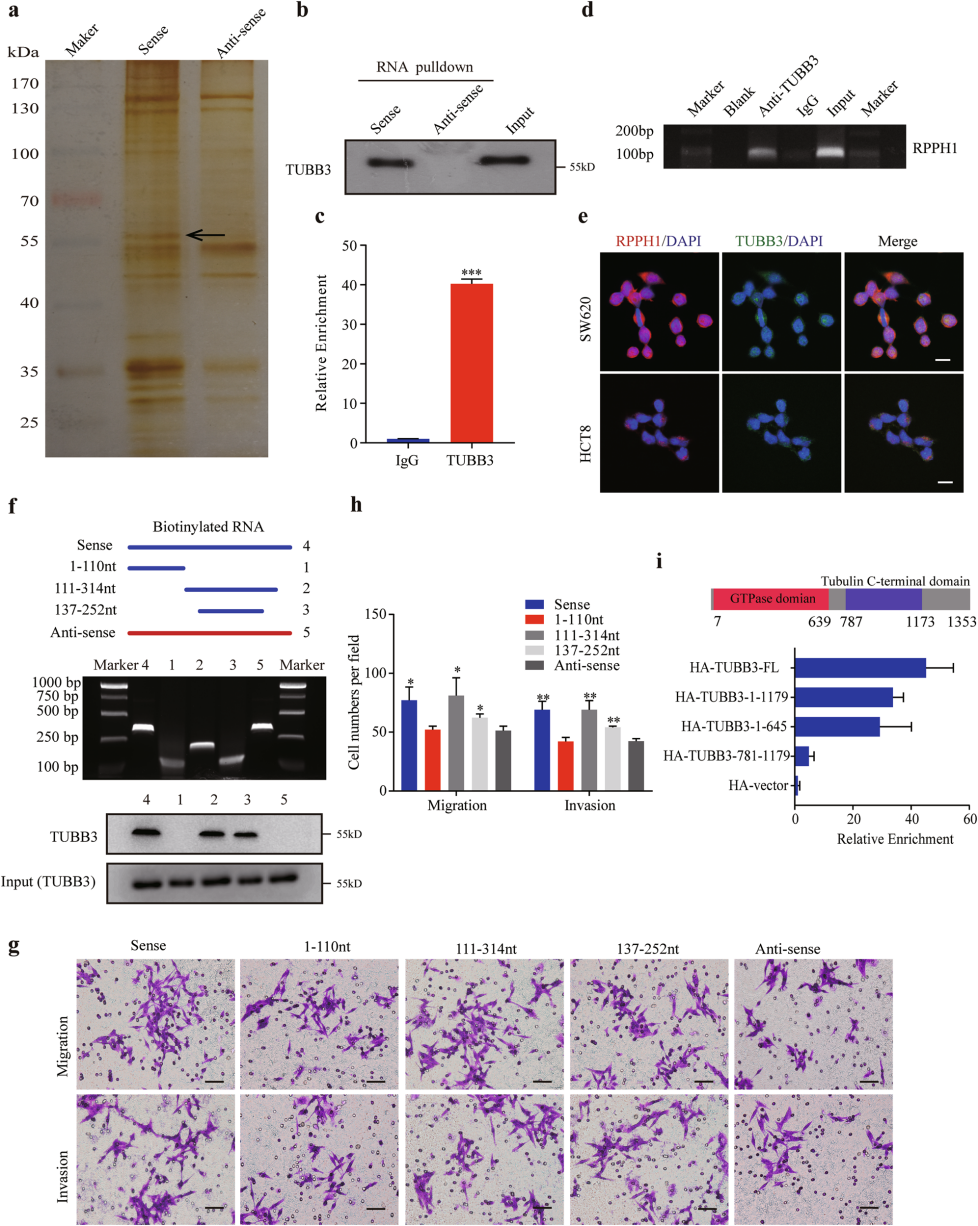
*RPPH1* physically interacts with TUBB3 in CRC cells. **a** Biotinylated sense and antisense *RPPH1* were transcribed in vitro and incubated with SW620 whole-cell lysates for RNA pulldown assays. A specific band (arrow) at about 55 kDa was excised after sliver staining. **b** Immunoblotting for specific associations of TUBB3 with RPPH1. **c**, **d** RNA immunoprecipitation (RIP) assays were performed using antibodies against TUBB3. qRT-PCR (**c**) and RT-PCR (**d**) assays were used to detect *RPPH1*. **e** Confocal FISH images demonstrated *RPPH1* co-localization with TUBB3 in the cytoplasm in SW620 and HCT8 cells. Scale bar = 10 μm. **f** Serial deletions of *RPPH1* were used to identify the domains of *RPPH1* that bond to TUBB3 by the RNA pulldown assays. **g**, **h** Transwell migration and invasion assays in stable *RPPH1* overexpression or truncated *RPPH1* SW620 cells. Scale bar = 50 μm. **i** RIP assays were used to identify the domains of TUBB3 that bind to *RPPH1* in SW620 cells. Values are represented as mean ± SD. *p < 0.05, **p *<* 0.01, and ***p < 0.001

Next, we performed RNA pulldown assays to identify the protein partners of *RPPH1* in order to uncover the molecular mechanism why *RPPH1* could promote CRC cells metastasis. One specific band appeared on the electrophoretic gel at approximately 55 kDa in contrast to the antisense *RPPH1* (Fig. 4a). Then the gel was subjected to mass spectrometry and we finally identified *RPPH1*-interacting protein TUBB3 according to protein mass and matched unique peptides (12/14). We then confirmed this finding by an independent immunoblot (Fig. 4b) and an RNA immunoprecipitation (RIP) assay (Fig. 4c, d). Moreover, we performed *RPPH1* and TUBB3 immunofluorescence and found the co-localization of *RPPH1* and TUBB3 in the cytoplasm of SW620 and HCT8 cells (Fig. 4e). After that, we constructed a series of biotin-labeled *RPPH1* fragments according to its secondary structure to determine which region binds to TUBB3 (Supplementary Fig. 3a). As shown in Supplementary Fig. 3a, region 2 contains region 3. Based on the RNA pulldown as well as immunoblotting experiments, region 2 bound more TUBB3 than region 3 (Fig. 4f). Migration and invasion assays with *RPPH1* fragments showed that both region 2 and region 3 could promote CRC cell migration and invasion. However, the capability to migrate through the membrane was demonstrated to be stronger in cells transfected with region 2 than those transfected with region 3 (Fig. 4g, h, Supplementary Fig. 3b, c). Moreover, the secondary structure of region 2 excluding region 3 could not be constructed in vitro, which precludes us from assessing this secondary structure’s function. Therefore, we hypothesize that the binding region of *RPPH1* to TUBB3 is region 2 (111–314 nt fragment). Meanwhile, we identified that the GTPase domain of TUBB3 was responsible for the interaction with *RPPH1* (Fig. 4i, Supplementary Fig. 3d). Taken together, these findings suggested that *RPPH1* physically binds to TUBB3 in CRC cells.

### *RPPH1* promotes CRC cell migration, invasion, and EMT via TUBB3

**Fig. 5:**
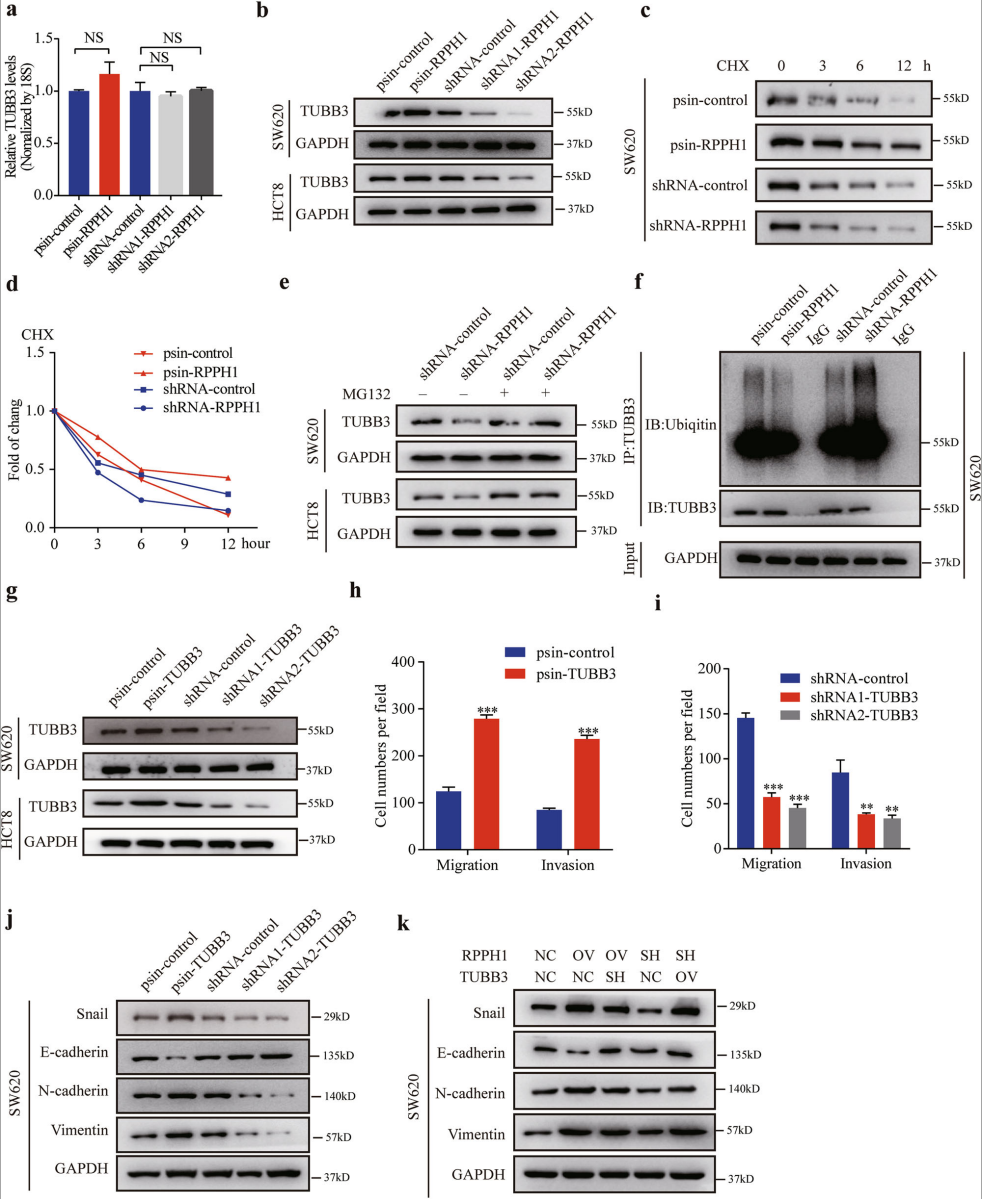
TUBB3 is the functional downstream target of *RPPH1* in CRC cells. **a** Relative TUBB3 mRNA levels were quantified by qRT-PCR in SW620 cells with stable RPPH1 overexpression or knockdown. 18S rRNA served as the control. **b** WB analysis for TUBB3 protein levels in stable *RPPH1* overexpression or knockdown SW620 and HCT8 cells. **c**, **d** SW620 cells with stable *RPPH1* overexpression or knockdown were treated with cycloheximide (CHX, 50 μg/ml) for the indicated times and TUBB3 protein levels were analyzed via WB analysis. **e** Stable *RPPH1* knockdown SW620 and HCT8 cells were treated with MG132 (25 μM) for 12 h and then TUBB3 protein levels were analyzed via WB analysis. **f** Cell lysates form stable *RPPH1* overexpression or knockdown SW620 cells treated with MG132 for 12 h were immunoprecipitated (IP) with either control IgG or TUBB3 antibody and then immunoblotted for ubiquitin and TUBB3. **g** WB analysis for TUBB3 protein levels in stable TUBB3 overexpression or knockdown SW620 and HCT8 cells. **h**, **i** Transwell migration and invasion assays in stable TUBB3 overexpression (**h**) or knockdown (**i**) HCT8 cells. **j** The EMT effect was validated by WB analysis of epithelial or mesenchymal markers in SW620 cells. **k** Rescue assays for WB analysis of the change of EMT markers were performed in SW620 cells with *RPPH1* and TUBB3 changing. Values are represented as mean ± SD. NS no significant. **p *<* 0.01, and ***p *<* 0.001

Previous studies have revealed that TUBB3 is overexpressed in CRC and is associated with EMT^28^. In our study, we found *RPPH1* promotes CRC cell metastasis and physically interacts with TUBB3. Hence, we wondered whether *RPPH1* performed this function via TUBB3. We identified *RPPH1* levels had no effect on TUBB3 mRNA levels in CRC cells (Fig. 5a, Supplementary Fig. 4a), whereas TUBB3 protein levels increased when *RPPH1* was overexpressed. In agreement with these data, the TUBB3 protein levels diminished when *RPPH1* was knocked down (Fig. 5b). We next treated SW620 and HCT8 cells with the protein synthesis inhibitor cycloheximide (CHX) and measured the stability of the TUBB3 protein. The protein stability increased when *RPPH1* was upregulated and decreased when *RPPH1* was silenced (Fig. 5c, d, Supplementary Fig. 4b, c). In addition, we proved that proteasome inhibitor MG132 attenuates this effect (Fig. 5e). As expected, the ubiquitination of TUBB3 increased when *RPPH1* was knocked down and decreased when *RPPH1* was overexpressed in SW620 (Fig. 5f) and HCT8 cells (Supplementary Fig. 4d). To determine whether TUBB3 can promote CRC cells migration, invasion, and EMT, we constructed TUBB3 overexpressed and knockdown CRC cell lines (Fig. 5g). Migration and invasion assays revealed that TUBB3 overexpression enhanced CRC cells migration and invasion abilities (Fig. 5h, i, Supplementary Fig. 4e, f). Consistently with these data, expression of mesenchymal markers N-cadherin and vimentin increased whereas epithelial markers E-cadherin diminished in western blotting when TUBB3 was upregulated. The TUBB3 knockdown had the opposite effect (Fig. 5j, Supplementary Fig. 4g). After that, we investigated whether *RPPH1* promotes migration, invasion, and EMT via TUBB3. When *RPPH1* was overexpressed, the TUBB3 knockdown reduced N-cadherin and vimentin levels but increased E-cadherin expression. TUBB3 attenuated the N-cadherin and vimentin downregulation when *RPPH1* was repressed (Fig. 5k, Supplementary Fig. 4h). Altogether, these results provide evidence that *RPPH1* promotes migration, invasion, and EMT via TUBB3.

### CRC cell-derived exosomal *RPPH1* induces macrophages M2 polarization in vitro

**Fig. 6:**
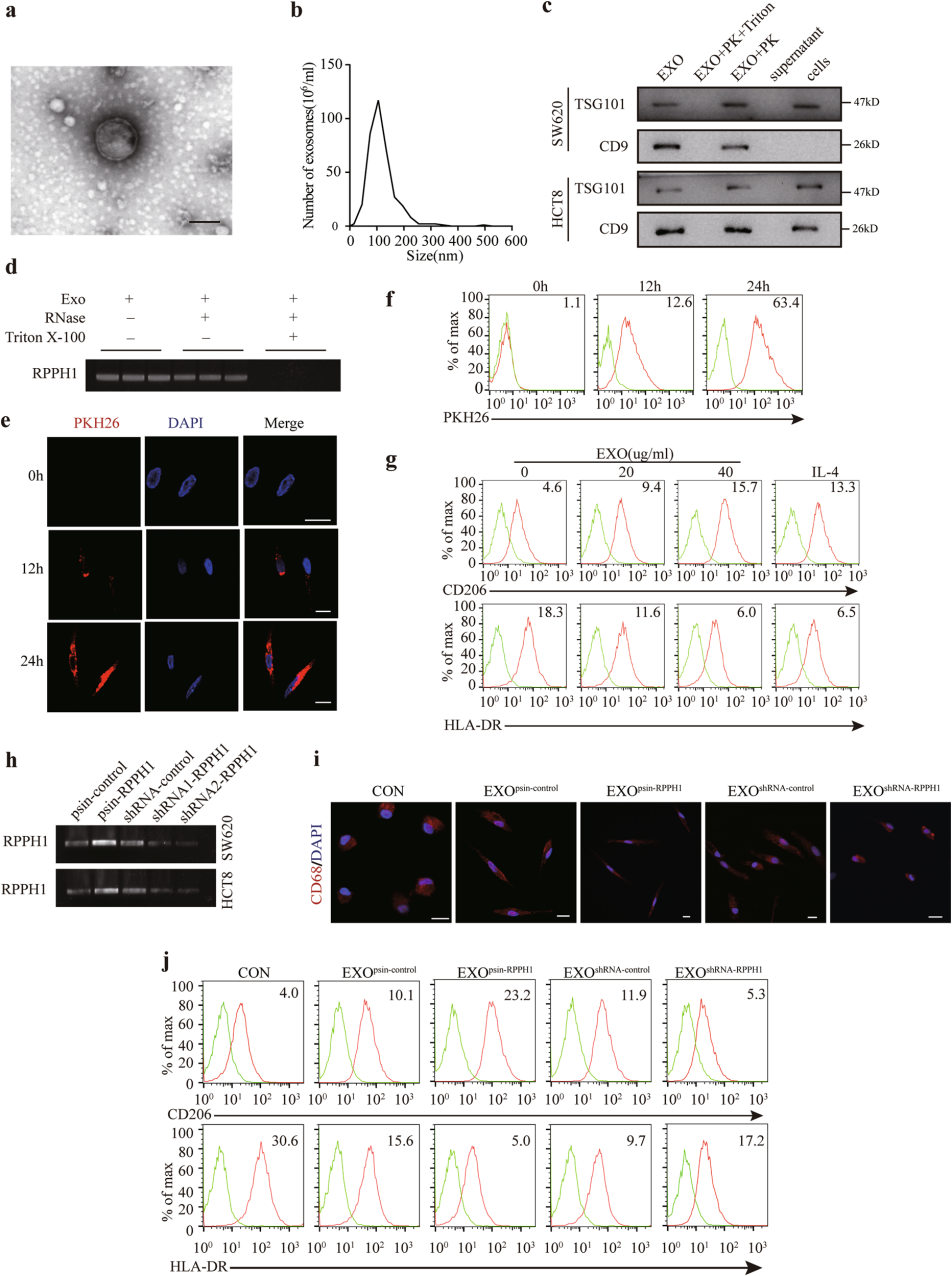
CRC cell-derived exosomal *RPPH1* induces macrophages M2 polarization. **a**, **b** Exosomes isolated from supernatants of CRC cells via ultracentrifugation were detected by transmission electron microscopy and Particle Metrix. Scale bar = 100 μm. **c** WB analysis of exosomes markers TSG101 and CD9. Exosomes extracts were treated with Triton X-100 + proteinase K or proteinase K alone. **d** PAGE analysis of *RPPH1* levels in exosomes after RT-PCR treated with RNase (2 mg/ml) alone or combined with Triton X-100 (0.1%) for 15 min. **e**, **f** Confocal microscopy and flow cytometric analysis of the internalization of PKH26-labeled exosomes in MDMs. Scale bar = 10 μm. **g** Flow cytometric analysis of the expressions of CD206/HLA-DR in macrophages treated with different concentrations of exosomes isolated from supernatants of CRC cells. Numerical values denote the relative fluorescence intensity. **h** PAGE analysis of *RPPH1* levels in exosomes isolated from supernatants of stable *RPPH1* overexpression or knockdown cells. **i** Confocal microscopy of the macrophages treated with exosomes with different *RPPH1* levels. Scale bar = 10 μm. **j** Flow cytometric analysis of the expressions of CD206/HLA-DR in macrophages treated with exosomes with different RPPH1 levels. Numerical values denote the relative fluorescence intensity

Intriguingly, we found *RPPH1* is abundant in blood exosomes of 12 CRC patients according to the exoRBase database (Supplementary Fig. 5a)^29^. Therefore, we further explored the underling mechanism of the potential link between *RPPH1* with tumor environment. Before the exosomes isolation, we performed terminal deoxynucleotidyl transferase dUTP nick end labeling (TUNEL) assay to evaluate the cell condition and eliminate apoptotic bodies or random cell debris (Supplementary Fig. 5b). Next, we isolated the exosomes from supernatants of CRC cells and identified them via transmission electron microscopy (TEM), Particle Metrix (PMX) and western blotting through ultracentrifugation. As presented in Fig. 6a, b, the typical particles were about 100 nm in diameter. We next identified the presence of exosomal markers TSG101 and CD9 (Fig. 6c). These data indicated we successfully isolated CRC cell exosomes. We next performed RT-PCR and polyacrylamide gel electrophoresis (PAGE) to confirm the existence of *RPPH1* in exosomes. When the exosomes were treated with RNase, *RPPH1* levels remained unchanged. However, when the samples were treated with RNase and Triton X-100, *RPPH1* levels dramatically diminished (Fig. 6d). Altogether, we proved that *RPPH1* was mainly packaged in exosomes.

Exosomes play a vital role in the cell communication by transporting lncRNAs^18^ and TAMs are the most abundant cells in tumor microenvironment^20,21^. Therefore, we hypothesized that exosomal *RPPH1* strongly involved in the communication between cancer cells and TAMs. First, to explore whether exosomes could be internalized into macrophages, PKH26-labeled exosomes were incubated with human monocyte-derived macrophages (MDMs) for 12 or 24 h and then confocal microscopy (Fig. 6e) and flow cytometric analysis (Fig. 6f) revealed rapid uptake of exosomes by MDMs. For functional assays, MDMs were treated with 20 or 40 μg/ml exosomes (20 ng/ml IL-4 treatment as a positive control) for the indicated times. In response to exosomes stimulation, these cells exhibited a CD206^high^/HLA-DR^low^ phenotype (Fig. 6g), with a stretched and elongated cellular morphology (Supplementary Fig. 5c). In comparation with untreated MDMs, MDMs treated with exosomes expressed significantly more M2 macrophages markers CCL17, CCL18, CXCL8, IL-10, and TGF-β, but there were no significant changes of M1 macrophage markers TNF-α, IL-6, and IL-1b via qRT-PCR (Supplementary Fig. 5d). These findings verified that CRC cell exosomes promoted macrophages M2 polarization. Then we isolated the exosomes from the supernatants of *RPPH1* overexpressed or knockdown CRC cells and detected the exosomal *RPPH1* levels were consistent with these changes in the parental cells (Fig. 6h). The macrophages exhibited a CD206^high^/HLA-DR^low^ phenotype, M2 morphology, and upregulation of M2 markers, when treated with exosomes with higher *RPPH1* levels. As expected, treatment with exosomes with lower *RPPH1* levels increased the expression of M1 markers and decreased M2 markers (Fig. 6i, j, Supplementary Fig. 5e). Overall, CRC cell-derived exosomal RPPH1 promotes macrophages M2 polarization in vitro.

### Exosomal *RPPH1* mediates macrophages M2 polarization to promote CRC cell metastasis and proliferation in vivo

**Fig. 7:**
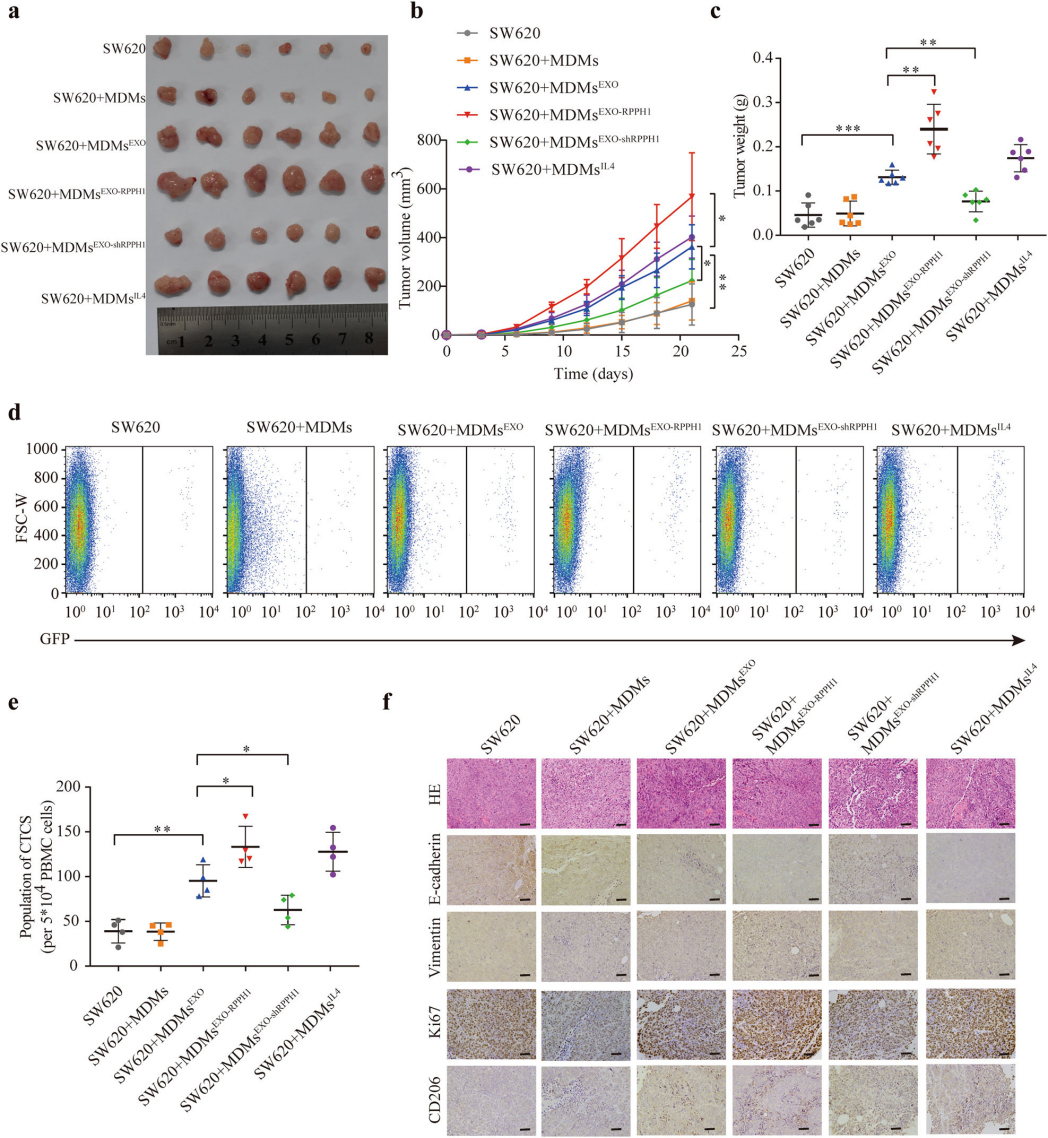
Exosomal *RPPH1* mediated M2 macrophages polarizing to promote CRC cells metastasis and proliferation in vivo. **a** Tumors in nude mice subcutaneously injected with SW620 and different *RPPH1* levels exosomes or IL-4-treated MDMs. *n* = 6 per group. **b** Tumor volumes were measured in different groups every 3 days. *n* = 6 per group. **c** Tumor weight was measured 3 weeks later when the mice were sacrificed. *n* = 6 per group. **d** Flow cytometric analysis was performed to analyze the GFP-labeled CTCs from whole blood when the mice were sacrificed. **e** Statistics of the flow cytometric analysis. *n* = 4 per group. **f** H&E staining and immunohistochemistry of E-cadherin, Vimentin, Ki67, and CD206. Scale bar = 50 μm. Values are represented as mean ± SD. *p *<* 0.05, **p *<* 0.01, and ***p *<* 0.001

To clarify the effect of exosomal *RPPH1*-mediated macrophages M2 polarization on CRC cells, tumor cells labeled with green fluorescent protein (GFP) and MDMs treated with exosomes containing different *RPPH1* levels or with IL-4 were subcutaneously injected into nude mice (*n* = 6 per group). We measured the tumor volumes every 3 days. We found that MDMs treated with exosomes containing higher *RPPH1* levels increased the volume and weight of tumors (Fig. 7a–c). Three weeks later, flow cytometric analysis was conducted to analyze circulating tumor cells (CTCs) from whole-blood samples. As expected, MDMs treated with exosomes containing higher *RPPH1* levels increased the number of CTCs as compared to the control group. Conversely, MDMs treated with exosomes containing lower *RPPH1* levels decreased the number of CTCs (Fig. 7d, e). To further demonstrate the effect of exosomal *RPPH1-*mediated macrophages M2 polarization on CRC cells, we examined the protein levels of E-cadherin, Vimentin, Ki67, and CD206 in the xenografts by immunohistochemical analysis (IHC). Similarly, MDMs treated with exosomes containing higher *RPPH1* levels raised the levels of Vimentin and Ki67, but decreased the expression of E-cadherin in the xenografts (Fig. 7f). In summary, *RPPH1* promoted CRC cells metastasis and proliferation via mediating macrophages M2 polarization.

### Circulating exosomal *RPPH1* may be a diagnostic biomarker of CRC

**Fig. 8:**
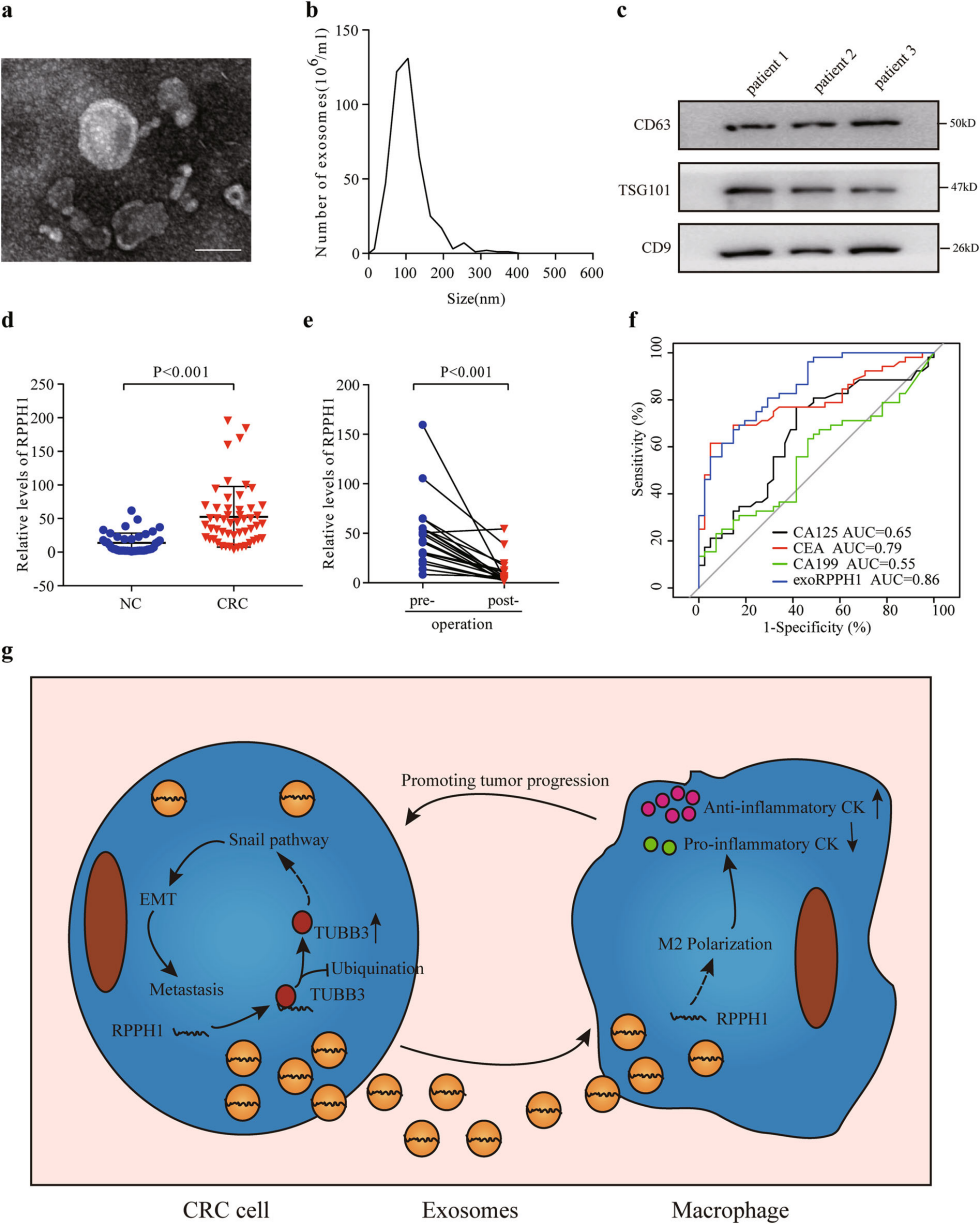
Circulating exosomal *RPPH1* could be a potential diagnosis biomarker for CRC. **a**, **b** Exosomes isolated from CRC patients’ plasma samples by Exosome RNA Amplification Kit (SBI) were detected by transmission electron microscopy and Particle Metrix. Scale bar = 100 μm. **c** WB analysis of exosomes markers CD63, TSG101, and CD9. **d** Relative expression of exosomal *RPPH1* in CRC patients (*n* = 52) and healthy donors (*n* = 41) via qRT-PCR. Exogenous λ polyA RNA served as the control. **e** Levels of exosomal *RPPH1* in CRC patients’ plasma changes before surgery (pre-operation) and 30 days after tumor resection (post-operation). **f** ROC curves for detection of CRC using exosomal *RPPH1*, CA125, CEA, or CA199. **g** A schematic model of *RPPH1* functions in tumor metastasis

To further explore the clinical significance of exosomal *RPPH1*, we isolated exosomes from CRC patients’ and healthy donors’ plasma samples. TEM and PMX analysis revealed the size and shape of vesicles from plasma was consistent with that of exosomes (Fig. 8a, b). Moreover, we performed western blots and detected exosomal markers CD63, TSG101, and CD9 (Fig. 8c). Then, we determined the exosomal *RPPH1* levels in plasma samples of 52 CRC patients and 41 healthy donors by qRT-PCR. We found the exosomal *RPPH1* levels were significantly higher in CRC patients than healthy donors (Fig. 8d). Besides, we measured the exosomal *RPPH1* levels in 20 CRC patients 3 months after tumor resection. We found the exosomal *RPPH1* levels dramatically decreased in post-operative samples in comparison with their paired pre-operative samples (Fig. 8e). Finally, we compared the diagnostic power between exosomal *RPPH1* and the conventional (wildly accepted) tumor markers CEA, CA199, and CA125 via receiver operating characteristic (ROC) curve analyses. As shown in Fig. 7f, the area under ROC curve (AUC) for exosomal *RPPH1*, CEA, CA199, and CA125 was 0.856 (95% CI = 0.783–0.930, *p* *<* 0.001), 0.790 (95% CI = 0.698–0.882, *p* < 0.001), 0.544 (95% CI = 0.437–0.672, *p* = 0.371), and 0.654 (95% CI = 0.540–0.767, *p* = 0.371) respectively. These data indicated that exosomal *RPPH1* was superior to the traditional tumor markers. Altogether, circulating exosomal *RPPH1* is a good diagnostic biomarker for CRC.

## Discussion

Metastasis of CRC is a major contributing of poor prognosis^30^. LncRNAs have been reported to participate in diverse biological processes, including metastasis^10,31^. In the present study, we found that lncRNA *RPPH1* which is associated with advanced TNM stage and poor prognosis. Consistently, *RPPH1* enhanced CRC cells migration and invasion in vitro and in vivo. Mechanistically, *RPPH1* binds to TUBB3 to prevent its ubiquitination and then induces EMT in CRC cells. Furthermore, we found *RPPH1* can be transmitted by exosomes to macrophage to mediate macrophage M2 polarization, thereby promoting CRC cell metastasis and proliferation. These findings illustrate that *RPPH1* promotes CRC cell metastasis by functioning within cells and changing the tumor microenvironment.

TUBB3, β-III tubulin, is mainly expressed in neuronal tissues and testes^32^. Recent studies revealed that TUBB3 is overexpressed in solid tumors, such as breast, ovarian, testis, and colorectal cancer^28,33^. Furthermore, TUBB3 overexpression is a marker of taxane- or vinca-alkaloid-based drug resistance and is associated with poor outcome in various epithelial tumors, including CRC^34–36^. In this study, we explained why TUBB3 is overexpressed in CRC. *RPPH1* binds to TUBB3 to enhance its stability and prevent its ubiquitination. Previous study has found TUBB3 can modulate the behavior of Snail overexpressed cells^28^. In our study, we also proved TUBB3 can activate the Snail pathway, thus promoting EMT. TUBB3 facilitates the transport and proper localization of N-cadherin to the plasma membrane^37^, but how TUBB3 activates the Snail pathway or promotes EMT remain largely unknown.

Our study offers an important finding that *RPPH1* can be transported by exosomes to macrophages to promote their polarization. TAMs are the most abundant cells in tumor microenvironment^20,21^. Epidemiological studies suggest that TAMs density has a strong association with the poor prognosis in various tumors^22,23^. TAMs promote tumor growth and metastasis mostly by secreting tumor-promoting factors, such as inflammatory cytokines^38,39^. In this study, we established that exosomes isolated from CRC cells supernatants can be internalized by macrophages and exosomal *RPPH1* exerts an important influence on this process. Moreover, we demonstrated that the levels anti-inflammatory cytokines increased and those of pro-inflammatory cytokines decreased when macrophages were treated with exosomes. The number of CTCs increased as compared to the control. Furthermore, we found TAMs can promote tumor metastasis and growth in an animal experiment. Even though we observed *RPPH1* had no effect on CRC cells proliferation in vitro, *RPPH1* could be transmitted by exosomes to promote macrophages M2 polarization to promote CRC cells proliferation.

Early diagnosis and treatment of CRC can improve the prognosis and OS significantly^40^. However, there is no effective biomarker for the early diagnosis of CRC. As is known to all, CEA is the most convincing biomarker at present. Nevertheless, CEA has low sensitivity and specificity, especially in the early stage diagnosis. Numerous studies suggest that tumor cells can secrete exosomes containing lncRNAs into body fluids, such as blood^41,42^. Hence, exosomal lncRNAs could be meaningful biomarkers for the diagnosis. Our study indicates that the exosomal *RPPH1* level is higher in the plasma of CRC patients relative to healthy donors. Meanwhile, the *RPPH1* level significantly decreased after tumor resection. In addition, we compared the plasma exosomal *RPPH1* with the traditional biomarkers, such as CEA, CA199, and CA125. The AUC for exosomal *RPPH1* was 0.856, which is better than the AUCs of the traditional biomarkers. This means plasma exosomal *RPPH1* exhibits higher sensitivity and specificity. Moreover, we believe that plasma exosomal *RPPH1* can be a new promising biomarker for the diagnosis and prognosis biomarker for CRC.

In summary, we provided the evidence that *RPPH1* overexpression is associated with poor CRC prognosis. *RPPH1* promotes CRC cells metastasis by binding to TUBB3, thus inhibiting its ubiquitination and enhancing exosomes-mediated macrophages M2 polarization and influences the tumor microenvironment (Fig. 7g). Moreover, plasma exosomal *RPPH1* may be a diagnostic marker and a therapeutic target in CRC.

## Material and methods

### Cell lines and cell cultures

The human CRC cell lines HCT8, SW620, HT29, and human embryonic kidney 293T cells were purchased from American Type Culture Collection (ATCC). All of the cells were cultured at 37 °C in Dulbecco’s modified Eagle's medium (DMEM; Gibco, Thermo Fisher Scientific, St Peters, MO, USA) supplemented with 10% fetal bovine serum (FBS; Gibco, Thermo Fisher Scientific, St Peters, MO, USA) in a 5% CO_2_ atmosphere.

### Patients and samples

Sixty-one paired human CRC samples and their matched NATs were used to analyze RPPH1, E-cadherin, and Vimentin RNA levels; these tissue samples were collected from the Sixth Affiliated Hospital of Sun Yat-sen University, Guangzhou, China. The matched NATs were obtained at least 3 cm away from the tumor. All the patients did not receive chemotherapy or radiotherapy before surgery. All tissues upon resection were immediately frozen in liquid nitrogen and stored at a −80 °C refrigerator upon resection until further use. All the patients signed informed consent. This study was approved by the Ethics Committee of the Sixth Affiliated Hospital, Sun Yat-sen University.

### RNA sequencing

Total RNA was extracted from seven paired CRC samples and NATs from CRC patients with liver metastasis. The RNA purity was analyzed on a Bioanalyzer 2200 instrument (Aligent). Then the RNA was treated with RiboMinus Eukaryote Kit (Qiagen, Valencia, CA) to remove ribosomal RNA and a cDNA library was constructed. Finally deep sequencing was performed with an Illumina HiSeq 3000 (Illumina, San Diego, CA). In the screening, when the fold change > 2 with a *p* value < 0.05, the lncRNA was identified as an upregulated lncRNA. On the contrary, it was identified as a downregulated lncRNA when the fold change < 0.5 with a *p* value < 0.05. The NGS data used in the study (GSE138202) are available in a public repository from NCBI.

### Vectors construction and construction of stable cell lines

Pfu Ultra II Fusion HS DNA Polymerase (Stratagene, Agilent Technologies) was used to amplify the cDNA encoding RPPH1 or TUBB3 and then the cDNA was cloned into lentiviral expression vector pSin-EF2-Pur reformed from pSin-EF2-Sox2-Pur (Addgene, Cambridge, MA, USA). The oligonucleotides to suppress RPPH1 or TUBB3 expression were designed by RiboBio (Guangzhou, China). Then they were cloned into lentiviral expression vector pLKO.1-Pur (Addgene, Cambridge, MA, USA). The plasmids were verified by sequencing. Empty vector pSin-EF2-Pur and vector pLKO.1-Pur carrying a scrambled shRNA served as a control. 293T cells were incubated with the vectors described above, psPAX2 and pMD2G (Addgene) according to the manufacturer’s instructions. The supernatant containing infectious lentivirus was filtered through 0.22 μm PVDF filters after harvesting at 24 h post transfection and then added into the plate to infect SW620 and HCT8 cells. Infection efficiency was confirmed by qRT-PCR. The methods for transfection and lentiviral infection were described in a previous study^43^. The oligonucleotide sequences for vector construction are listed in Supplementary Table 5.

### RNA pulldown assay

Biotin-labeled full-length RPPH1 and antisense RPPH1 were synthesized in vitro with the Transcript Aid T7 High Yield Transcription Kit (Thermo Scientific)^44^. Then the MEGAclear^TM^ Kit (Thermo Scientific) was employed to recycle the sequences according to the manufacturer’s instructions. The sequences were incubated with cell lysates at room temperature for 4 h, and then the biotin-labeled RNAs with their binding protein partner were pulled down by streptavidin magnetic beads (Thermo, USA) at 4 °C overnight. The proteins were separated by electrophoresis and visualized with the Coomassie Blue Staining Kit (Beyotime, China). The different bands between sense and antisense RPPH1 were identified using mass spectrometry and retrieved in human proteomic library. The oligonucleotide sequences for the RNA pulldown are listed in Supplementary Table 6.

### RNA ISH and IHC scoring

We evaluated the marker staining results according to the previous study^45^. The staining intensity was graded 4 stages: 0 (none), 1 (weak), 2 (moderate), and 3 (strong). The percentage of expression was graded 5 stages: 0 (<5% staining), 1 (5–25% staining), 2 (25–50% staining), 3 (50–75% staining), and 4 (>75% staining). The sum of both scores served as the final score. Two pathologists performed the scoring analyses according to the above criteria. Those two pathologists were blinded to this study.

### Exosome extraction and identification

Ultracentrifugation methods were applied to isolate exosomes from the supernatants of SW620 and HCT8 cells as previous study^46^. In brief, the cells were cultured in a complementary medium until about 80% confluence, and then the medium was replaced the defined medium without FBS. After 2 days of culture, we harvested the supernatants and centrifuged them at 300 × *g* for 15 min, 2000 × *g* for 15 min, and 10,000 × *g* for 30 min. The supernatants were filtrated through a 0.22 μm PVDF filter (Millipore, USA). Then the supernatants were collected to isolate exosomes by ultracentrifugation at 120,000 × *g* for 70 min (Beckman Coulter) twice. Particle Metrix (PMX), transmission electron microscopy (TEM), and western blotting were used to identify the exosomes.

The plasma exosomes and exosome RNA were isolated by SeraMir™ Exosome RNA Amplification Kit (SBI) according to the manufacturer’s instructions. In brief, 500 μl of plasma was used to combine with 120 μl ExoQuick incubated at 4 °C for 30 min and then we obtained the exosomes pellets by centrifuging at 13,000 r.p.m. for 2 min. The exosomes pellet was lysed with LYSIS Buffer, and next, we purified the exosome RNA. Plasma exosomes were also identified by PMX, TEM, and western blotting. The exosome RNA levels were normalized by exogenous λ polyA RNA (Takara, China) for qPCR^47^.

### Isolation and cultivation of monocytes/macrophages

Human peripheral blood monocytes (PBMs) were isolated from healthy volunteer donors by density-gradient centrifugation with Ficoll-Hypaque (Pharmacia, Peapack, NJ) as previously described^48,49^. PBMs were seeded at 2 × 10^6^/ml in 24-well plates in DMEM medium (GIBCO) with 10% heat-inactivated human AB serum (Gemini Bio- Products, West Sacramento, CA), 50 U of penicillin per ml, 50 μg of streptomycin per mL, 2 mM l-glutamine, and 100 ng/ml human M-CSF. By repeated gentle washing with a warm medium and replacement of supernatants, nonadherent cells were removed after 5 days of culture. After that, the macrophages were treated with 20 ng/ml recombinant IL-4 (Peprotech) or CRC cells exosomes for one day. The macrophages surface maker CD206 (eBioscience), CD68 (eBioscience), and HLA-DR (eBioscience) were analyzed by flow cytometry (BD Biosciences, New Jersey, USA).

### Animal experiments

The animal experiments were approved by the Institutional Animal Care and Use Committee of Sun Yat-sen University, Guangzhou, China. Male BALB/c nude mice (4–5 weeks old) were purchased from the Animal Experiment Center of Sun Yat-Sen University and were randomized into control and experimental groups. Subcutaneous tumor growth assays (*n* = 6 per group) were performed as previously study^50^. Subcutaneous tumors were subjected to IHC. We analyzed the GFP-positive CTCs in PBMCs of mice by flow cytometry as the previously study^12^. A tail vein injection model was used for lung colonization assays (*n* = 6 per group). The investigator was blinded to the group allocation.

### Statistics analysis

GraphPad Prism Software (GraphPad Software, La Jolla, CA, USA) was used to perform statistics analysis. Two-tailed Student’s test, ANOVA with Tukey’s multiple comparisons post-test, and Pearson’s correlation analysis were performed for statistical comparisons. All statistics analysis data are expressed as mean ± standard error of the mean. All *p* values were two-sided and a *p* value < 0.05 was considered statistically significant. All experiment was performed at least three times.

The detailed methods of the procedures are provided in Supplementary methods.

## Supplementary information

Detailed Attribution of Authorship

Supplementary methods

Supplementary Figure Legends

Supplementary Figure 1

Supplementary Figure 2

Supplementary Figure 3

Supplementary Figure 4

Supplementary Figure 5

Supplementary Table 6

Supplementary Table 1

Supplementary Table 2

Supplementary Table 3

Supplementary Table 4

Supplementary Table 5
