# Correction: LncRNA *RPPH1* promotes colorectal cancer metastasis by interacting with TUBB3 and by promoting exosomes-mediated macrophage M2 polarization

**DOI:** 10.1038/s41419-020-2661-3

**Published:** 2020-06-16

**Authors:** Zhen-xing Liang, Hua-shan Liu, Feng-wei Wang, Li Xiong, Chi Zhou, Tuo Hu, Xiao-wen He, Xiao-jian Wu, Dan Xie, Xian-rui Wu, Ping Lan

**Affiliations:** 1 Department of Colorectal Surgery, The Sixth Affiliated Hospital, Sun Yat-sen University, Guangzhou, Guangdong China; 2 Guangdong Provincial Key Laboratory of Colorectal and Pelvic Floor Diseases, The Sixth Affiliated Hospital, Sun Yat-sen University, Guangzhou, Guangdong China; 3 Guangzhou Regenerative Medicine and Health Guangdong Laboratory, Guangzhou, China; 4 State Key Laboratory of Oncology in South China, Collaborative Innovation Center for Cancer Medicine, Sun Yat-sen University Cancer Center, Guangzhou, Guangdong China; 5 Department of Endocrinology, The First Affiliated Hospital of Sun Yat-sen University, Guangzhou, China

**Keywords:** Cancer microenvironment, Colorectal cancer

Correction to: *Cell Death & Disease*

10.1038/s41419-019-2077-0

published online 04 November 2019

Since online publication of this article, the authors noticed that there was an error in Supplementary Fig. 5. The label ‘exoRBase’ was missed from Fig. 5a, and an incorrect image was used in the compilation of Fig. 5c. The corrected image is provided below. The authors confirm that the errors do not affect the results or conclusions of the study, and apologise for any inconvenience caused.



