# Supplementary material for: LncRNA RPPH1 promotes colorectal cancer metastasis by interacting with TUBB3 and by promoting exosomes-mediated macrophage M2 polarization
Source: Cell Death Dis. 2019 Nov 4;10(11):829. doi: 10.1038/s41419-019-2077-0 (PMC6828701; doi:10.1038/s41419-019-2077-0)
Supplement: Supplementary file 2 — Supplementary methods [file 41419_2019_2077_MOESM2_ESM.docx]

**Supplementary methods**

**RNA extraction and real-time PCR.**

The TRIzol Reagent (Thermo Fisher Scientific, St Peters, MO, USA) was employed to isolate total RNA from cells or tissue samples. The NanoDrop ND-2000 spectrophotometer (Thermo Fisher Scientific, St Peters, MO, USA) was used to determine the RNA concentrations and purity. We utilized the ReverTra Ace qPCR RT Kit (Toyobo Biochemicals, Kita-ku, Osaka, Japan) to perform reverse transcription according to the manufacturer’s instructions. The Applied Biosystems 7500 Sequence Detection system was used to carry out quantitative real-time reverse transcription PCR (qRT-PCR) with the SYBR Green PCR Master Mix (Applied Biosystems, Foster City, CA, USA). We generated standard curves and applied the 2^−△△CT^ method with normalized to 18S rRNA. We next used the gene of human hypoxanthine-guanine-phosphoribosyltransferase (hHPRT) to quantify cancer metastasis in mouse lungs. All the gene-specific primers were obtained from Invitrogen and the oligonucleotide sequences are listed in Supplementary Table 3.

**5’ and 3’ Rapid amplification of cDNA ends (RACE) analysis.**

The SMARTer RACE 5’ /3’ kit (Clonetech) was employed to perform the RACE analysis according to the manufacturer’s instructions with 1μg of total RNA. The gene-specific primers (GSP) and nested gene-specific primers (NGSP) designed according to the manual guidelines are listed in Supplementary Table 4.

**Western blotting.**

Radio-immunoprecipitation assay buffer (RIPA) consisting of 150 mM NaCl, 1% Triton X-100, 0.5% sodium deoxycholate, 0.1% of SDS, 50 mM Tris (pH 8) and protease inhibitors cocktail (Promega, Fitchburg, WI, USA) was applied to lyse cells and exosomes samples. Sodium dodecyl sulfate-polyacrylamide gel electrophoresis (SDS-PAGE) was carried out to separate the proteins and then all the proteins were transferred to polyvinylidene fluoride (PVDF) by the Trans-Blot System (Bio-Rad, CA, USA). Then the membranes were incubated with specific primary antibodies against E-cadherin (CST, #3195), N-cadherin (CST, #13116), Vimentin (CST, #5741), Snail (CST, #3879), TUBB3 (CST, #5568), GAPDH (Abcam, ab181602), CD9 (Abcam, ab92726), CD63 (Abcam, ab217345) and TSG101 (Abcam, ab125011) at 4℃ overnight after the membranes were blocked with 5% w/v skim milk (BD Biosciences, San Jose, CA, USA) in Tris-buffered saline with 0.1% v/v Tween 20 (TBST) at room temperature for 1h. Then the membranes were washed with TBST three times and incubated with a specific secondary antibody at room temperature for 1h. ECL Blotting Detection Reagents (Amersham Biosciences, Little Chalfont, Buckinghamshire, UK) were used to visualize the specific bands. GAPDH served as a control for western blots.

**Cell migration and invasion assays.**

The 24-well plate with 8-μm pore size chamber inserts (Corning) was used to carry out cell migration and invasion assays, and 5×10^4^ cells were seeded in the upper chamber well with the non-coated membrane (Millipore, Burlington, MA, USA) for migration assays. 1×10^5^ cells were seeded in the upper chamber well with the Matrigel-coated membrane (Millipore, Burlington, MA, USA) for invasion assays. 200 μl of DMEM without FBS was used to resuspend the cells and was added into the upper chamber well. Next, 800 μl of DMEM with 10% FBS was added into the lower chamber. After incubation at 37°C for 24 h, cells migrating through the membrane were stained with 4% paraformaldehyde for 15 min, and then stained with 0.1% crystal violet for 15 min. An inverted microscope (DMI4000B, Leica, Wetzlar, Germany) was utilized to image the cells and software ImageJ (ImageJ 1.46r, Bethesda, MD, USA) was used to quantify the cells.

**RNA Immunoprecipitation (RIP).**

The Magna RIP™ RNA-Binding Protein Immunoprecipitation Kit (Millipore, USA) was used to perform RIP experiments. According to the manufacturer’s instructions, 1 ×10^7^ cells were lysed with RIP lysis buffer and then co-immunoprecipitated with 5ug antibody against TUBB3 (Cell Signaling Technology). Normal rabbit IgG (Cell Signaling Technology) served as a negative control. The retrieved RNA was detected by qRT-PCR. The primers for measuring the relative level of RPPH1 are listed in Supplementary Table 3.

**RNA fluorescence in situ hybridization (FISH).**

According to the manufacturer’s instructions, the FISH kit (Ribo Bio, Guangzhou, China) was utilized to perform FISH in cells and the results are visualized by confocal microscopy laser-scanning microscope (Leica TCS-SP8, Leica Microsystems Inc, Buffalo Grove, IL, USA). CRC cells were fixed with 4% paraformaldehyde at room temperature for 10 min, treated with 0.5% Triton X-100 in phosphate buffer saline (PBS) at 4°C for 5 min and then pre-hybridized by Pre-hybridization Buffer at 37°C for 30 min. Finally the cells were hybridized with 2.5 ul 20uM CY3-labeled U6, 18S, and RPPH1 FISH probes (Ribo Bio) overnight at 37°C.

**RNA in situ hybridization (ISH).**

According to the manufacturer’s instructions, the ISH Detector kit (BSTER, Wuhan, China) was used to perform ISH in paraffin-embedded CRC tissues. CRC tissues were fixed with 4% paraformaldehyde at room temperature for 10 min; then they were digested with proteinase K at 37°C for 2 min and pre-hybridized at 37°C for 3 h. The tissues were hybridized with double 5’-3’-digoxin (DIG)-labeled RPPH1 ISH probes (TSINGKE Biological Technology) overnight at 37°C. Finally, tissues were incubated with an anti-digoxin monoclonal antibody conjugated with alkaline phosphatase and then incubated with 3, 3’-diaminobenzidine (DAB).

**Immunohistochemistry (IHC).**

Dimethylbenzene was applied to deparaffinize paraffin-embedded tissues followed by antigen retrieval. The tissues were blocked with normal goat serum at 37°C for 30 min. Next, the tissues were incubated with specific primary antibodies against E-cadherin (Cell Signaling Technology), N-cadherin (Cell Signaling Technology), Vimentin (Cell Signaling Technology), Ki 67 (Cell Signaling Technology) and TUBB3 (Cell Signaling Technology) at 4°C overnight. Finally, tissues were incubated with appropriate secondary antibodies and then incubated with 3, 3’-diaminobenzidine (DAB).

**Immunofluorescence assays (IF).**

The cells or tissues were incubated with specific primary antibody against E-cadherin (Cell Signaling Technology), Vimentin (Cell Signaling Technology), TUBB3 (Cell Signaling Technology) and CD68 (Cell Signaling Technology) at 4℃ overnight. Then they were incubated with Alexa488 or Alexa594-conjugated goat antibodies (Thermo Fisher Scientific, St Peters, MO, USA) against mouse or rabbit IgG for immunofluorescent staining. Finally, the samples were counterstained with DAPI and imaged with confocal laser-scanning microscope (Leica TCS-SP8, Leica Microsystems Inc, Buffalo Grove, IL, USA).

**Flow cytometry analysis.**

Cells were incubated with specific fluorochrome-conjugated antibodies and isotype-matched control IgG at room temperature for 30 min. Then this analysis was performed via flow cytometry (BD Biosciences, New Jersey, USA). The software FlowJo v10.0.7 was used to analyze the data.

**Exosome labeling and tracking.**

CRC cells exosomes were labeled with PKH26 Red Fluorescent membrane linker dye (Sigma-Aldrich) according to the manufacturer’s instructions. Then labeled exosomes were washed with PBS and centrifuged at 120,000×g for 70 min. Finally, the labeled exosomes were added into the supernatants of macrophages and the signals were detected by flow cytometry and confocal laser-scanning microscope.
