# Supplementary material for: LncRNA RPPH1 promotes colorectal cancer metastasis by interacting with TUBB3 and by promoting exosomes-mediated macrophage M2 polarization
Source: Cell Death Dis. 2019 Nov 4;10(11):829. doi: 10.1038/s41419-019-2077-0 (PMC6828701; doi:10.1038/s41419-019-2077-0)
Supplement: Supplementary file 3 — Supplementary Figure Legends [file 41419_2019_2077_MOESM3_ESM.docx]

**Supplementary Figure Legends**

**Supplementary Fig.1** ***RPPH1* is a non-coding RNA in CRC cells. a** and **b** Representative images of PCR products from the 5’ RACE (**a**) and 3’ RACE (**b**; left: Images of agarose gel electrophoresis analysis; right, sequencing of RACE products). **c** Sequence of *RPPH1* cloned from SW620 cells cDNA as shown. **d**, **e** and **f** The coding potential of *RPPH1* was analyzed via Coding potential Assessment Tool (CPAT) (**d**), Coding Potential Calculator (CPC) (**e**) and the PhyloCSF codon substitution frequency analysis (**f**). The regions above the baseline are predicted to be coding, while regions below the baseline are predicted to be noncoding. ACTB and GAPDH served as coding RNA controls, and HOTAIR and CCAT1 served as non-coding RNA controls.

**Supplementary Fig.2** ***RPPH1* promotes CRC cells migration, invasion and EMT *in vitro.* a** *RPPH1* relative expression levels in CRC cell-lines. **b** FISH analysis of the subcellular distribution of *RPPH1* in SW620 and HCT8 cells. Scale bar = 10 μm. **c** and **d** The qRT-PCR assays in HCT8 and SW620 cells with stable *RPPH1* overexpression (**c**) and knockdown (**d**). **e** and **f** Transwell migration and invasion assays in stable *RPPH1* overexpression (**e**) or knockdown (**f**) SW620 cells. Scale bar = 50 μm. **g** The MTT assays performed in SW620 cells to evaluate the effect of RPPH1 on cell proliferation. 18S rRNA served as the control in (**a**), (**c**) and (**d**). Values are represented as mean ± SD. **p* < 0.05, ***p* < 0.01 and ****p* < 0.001.

**Supplementary Fig.3 *RPPH1* physically interacts with TUBB3 in CRC cells. a** Second structure of *RPPH1* analyzed by RNAalifold WebServer，and the structured fragments are represented as numbered red boxed (1:+1-110nt; 2: 111-314nt; 3: 137-252nt). **b** and **c** Transwell migration and invasion assays in stable *RPPH1* overexpression or truncated *RPPH1* HCT8 cells. Scale bar = 100 μm. **d** RIP assays were used to identify the domains of TUBB3 that bind to RPPH1 in HCT8 cells. Values are represented as mean ± SD. ****p* < 0.001.

**Supplementary Fig.4 TUBB3 is the functional downstream target of *RPPH1* in CRC cells. a** Relative TUBB3 mRNA levels were quantified by qRT-PCR in HCT8 cells with stable RPPH1 overexpression or knockdown. 18S rRNA served as the control. **b** and **c** Stable *RPPH1* overexpression or knockdown HCT8 cells were treated with cycloheximide (CHX, 50 μg/ml) for the indicated times and TUBB3 protein levels were analyzed via WB analysis. **d** Cell lysates form stable RPPH1 knockdown HCT8 cells treated with MG132 for 12 h were immunoprecipitated (IP) with either control IgG or TUBB3 antibody and then immunoblotted for ubiquitin and TUBB3. **e** and **f** Transwell migration and invasion assays in stable TUBB3 overexpression (**e**) or knockdown (**f**) SW620 cells. **j** The EMT effect was validated by WB analysis of epithelial or mesenchymal markers in HCT8 cells. **k** Rescue assays for WB analysis of the change of EMT markers were performed in HCT8 cells with *RPPH1* and TUBB3 changing. Values are represented as mean ± SD. NS, no significant. **p*<0.05, ***p*<0.01, and ****p*<0.001.

**Supplementary Fig.5 CRC cell-derived exosomes induces macrophages Cytokine levels changes****. a** Heatmap of lncRNAs expression in blood exosomes of 12 CRC patients. **b** TUNEL assay was detected by Confocal microscopy. Right panel’s cells were positive control. Scale bar = 40 μm. **c** Confocal microscopy of the macrophages treated with different concentration exosomes. Scale bar = 10 μm. **d** The heatmap showed the cytokine levels changes in macrophages treated with different concentration exosomes via qRT-PCR. **e** CCL18, IL10 and TNFα levels in macrophages treated with exosomes with different *RPPH1* levels via qRT-PCR. Values are represented as mean ± SD. ***p*<0.01, and ****p* <0.001.
