# Supplementary material for: LncRNA RPPH1 promotes colorectal cancer metastasis by interacting with TUBB3 and by promoting exosomes-mediated macrophage M2 polarization
Source: Cell Death Dis. 2019 Nov 4;10(11):829. doi: 10.1038/s41419-019-2077-0 (PMC6828701; doi:10.1038/s41419-019-2077-0)
Supplement: Supplementary file 9 — Supplementary Table 6 [file 41419_2019_2077_MOESM9_ESM.docx]

**Supplementary Table 6. The oligonucleotide sequence for RNA pulldown**

| Name | Sequences |
| --- | --- |
| RPPH1-sense-F | TAATACGACTCACTATAGGGATAGGGAGACGGAGGGAAGCTCAT |
| RPPH1-sense-R | AATGGGCGGAGGAGAGTAGTC |
| RPPH1-antisense-F | ATAGGGCGGAGGGAAGCTCAT |
| RPPH1-antisense-R | TAATACGACTCACTATAGGGAGAAATGGGCGGAGGAGAGTAGTC |
