# Supplementary material for: LncRNA RPPH1 promotes colorectal cancer metastasis by interacting with TUBB3 and by promoting exosomes-mediated macrophage M2 polarization
Source: Cell Death Dis. 2019 Nov 4;10(11):829. doi: 10.1038/s41419-019-2077-0 (PMC6828701; doi:10.1038/s41419-019-2077-0)
Supplement: Supplementary file 10 — Supplementary Table 1 [file 41419_2019_2077_MOESM10_ESM.docx]

**Supplementary Table 1. The seven patients’ characteristics for next-generation sequencing**

| patient | Gender | Age | Pathological grade |
| --- | --- | --- | --- |
| CRC1 | female | 60 | IV |
| CRC2 | male | 51 | IV |
| CRC3 | male | 40 | IV |
| CRC4 | female | 56 | IV |
| CRC5 | male | 66 | IV |
| CRC6 | male | 57 | IV |
| CRC7 | female | 48 | IV |
