# Supplementary material for: LncRNA RPPH1 promotes colorectal cancer metastasis by interacting with TUBB3 and by promoting exosomes-mediated macrophage M2 polarization
Source: Cell Death Dis. 2019 Nov 4;10(11):829. doi: 10.1038/s41419-019-2077-0 (PMC6828701; doi:10.1038/s41419-019-2077-0)
Supplement: Supplementary file 11 — Supplementary Table 2 [file 41419_2019_2077_MOESM11_ESM.docx]

**Supplementary Table 2. Correlation between RPPH1 expression and clinicopathologic characteristics of CRC patients**

| Characteristics | Frequency (%) | RPPH1 expression level | | |
| --- | --- | --- | --- | --- |
|  |  | Low | High | *p*-value^a^ |
| Gender |  |  |  | 0.320 |
| Female | 130 | 78 | 52 |  |
| Male | 163 | 107 | 56 |  |
| Age |  |  |  |  |
| ≤58 | 136 | 93 | 43 | 0.083 |
| >58 | 157 | 92 | 65 |  |
| Stage |  |  |  | < 0.001 |
| I+II | 167 | 127 | 40 |  |
| II+IV | 126 | 58 | 68 |  |
| Metastasis |  |  |  | < 0.001 |
| + | 116 | 52 | 64 |  |
| - | 177 | 126 | 40 |  |

^a^ Chi-square test
