# Supplementary material for: LncRNA RPPH1 promotes colorectal cancer metastasis by interacting with TUBB3 and by promoting exosomes-mediated macrophage M2 polarization
Source: Cell Death Dis. 2019 Nov 4;10(11):829. doi: 10.1038/s41419-019-2077-0 (PMC6828701; doi:10.1038/s41419-019-2077-0)
Supplement: Supplementary file 12 — Supplementary Table 3 [file 41419_2019_2077_MOESM12_ESM.docx]

**Supplementary Table 3. The primers for real-time PCR**

| Gene | Forward primer | Reverse primer |
| --- | --- | --- |
| RPPH1 | CACTCCACTCCCATGTCCC | GTTCCAAGCTCCGGCAAA |
| GUSB | GACACGCTAGAGCATGAGGG | GGGTGAGTGTGTTGTTGATGG |
| 18S | CGGCTACCACATCCAAGGAA | GCTGGAATTACCGCGGCT |
| E-cadherin | GCCCCATCAGGCCTCCGTTT | ACCTTGCCTTCTTTGTCTTTGTTGGA |
| N-cadherin | TGTATGTGGGCAAGATCCACT | CTCGTCGATCAGGAAGATGGT |
| Vimentin | AGTCCACTGAGTACCGGAGAC | CATTTCACGCATCTGGCGTTC |
| TUBB3 | CTCGAGCCATTCTGGTGGAC | TTGGAGCAGGGGGAACTTAC |
| TGFβ | CAATTCCTGGCGATACCTCAG | GCACAACTCCGGTGACATCAA |
| CCL17 | AGGGACCTGCACACAGAGAC | CTCGAGCTGCGTGGATGTGC |
| CCL18 | CTTGTCCTCGTCTGCACCAT | CTTGTCCTCGTCTGCACCAT |
| CXCL8 | CACTGCGCCAACACAGAAAT | GCTTGAAGTTTCACTGGCATC |
| IL10 | CGAGATGCCTTCAGCAGAGT | CGCCTTGATGTCTGGGTCTT |
| TNFα | CCCATCTATCTGGGAGGGGT | GCGTTTGGGAAGGTTGGATG |
| IL6 | TCAATATTAGAGTCTCAACCCCCAA | CAGGGAGAAGGCAACTGGAC |
| IL1b | CAGAAGTACCTGAGCTCGCC | AGATTCGTAGCTGGATGCCG |
| HPRT | TTCCTTGGTCAGGCAGTATAATCC | AGTCTGGCTTATATCCAACACTTCG |
