# Supplementary material for: LncRNA RPPH1 promotes colorectal cancer metastasis by interacting with TUBB3 and by promoting exosomes-mediated macrophage M2 polarization
Source: Cell Death Dis. 2019 Nov 4;10(11):829. doi: 10.1038/s41419-019-2077-0 (PMC6828701; doi:10.1038/s41419-019-2077-0)
Supplement: Supplementary file 13 — Supplementary Table 4 [file 41419_2019_2077_MOESM13_ESM.docx]

**Supplementary Table 4. The primers for 5’ and 3’ Rapid amplification of cDNA end (RACE) analysis**

| Name | Sequences |
| --- | --- |
| RPPH1-5’RACE-GSP | GATTACGCCAAGCTTAATGGGCGGAGGAGAGTAGTCTGA |
| RPPH1-3’RACE-GSP | GATTACGCCAAGCTTATAGGGCGGAGGGAAGCTCATCAG |
| RPPH1-5’RACE-NGSP | GATTACGCCAAGCTTGTTCCAAGCTCCGGCAAAGGAGGCA |
| RPPH1-3’RACE-NGSP | GATTACGCCAAGCTTCACTCCACTCCCATGTCCCTTGGGA |
