# Supplementary material for: LncRNA RPPH1 promotes colorectal cancer metastasis by interacting with TUBB3 and by promoting exosomes-mediated macrophage M2 polarization
Source: Cell Death Dis. 2019 Nov 4;10(11):829. doi: 10.1038/s41419-019-2077-0 (PMC6828701; doi:10.1038/s41419-019-2077-0)
Supplement: Supplementary file 14 — Supplementary Table 5 [file 41419_2019_2077_MOESM14_ESM.docx]

**Supplementary Table 5. The primers for vector construction**

| Name | Sequences |
| --- | --- |
| RPPH1 F | CCCGGACGAATTCTTCGAAATAGGGCGGAGGGAAGCTCAT |
| RPPH1 R | TGCGGATCACTAGTGCTAGCAATGGGCGGAGGAGAGTAGTC |
| HA-TUBB3 F | CCCGGACGAATTCTTCGAAATGTACCCATACGATGTTCCAGATTACGCTATGAGGGAGATCGTGCACATC |
| HA-TUBB3 R | TGCGGATCACTAGTGCTAGCTCACTTGGGGCCCTGGGCCTC |
| HA-TUBB3 1-1179 F | CCCGGACGAATTCTTCGAAATGTACCCATACGATGTTCCAGATTACGCTATGAGGGAGATCGTGCACATC |
| HA-TUBB3 1-1179 R | TGCGGATCACTAGTGCTAGCGGCCTTGCGCCGGAACATGGC |
| HA-TUBB3 1-645 F | CCCGGACGAATTCTTCGAAATGTACCCATACGATGTTCCAGATTACGCTATGAGGGAGATCGTGCACATC |
| HA-TUBB3 1-645 R | TGCGGATCACTAGTGCTAGCGAGGGTGCGGAAGCAGATGTC |
| HA-TUBB3 781-1179 F | CCCGGACGAATTCTTCGAAATGTACCCATACGATGTTCCAGATTACGCTCCGCGCCTGCACTTCTTCATG |
| HA-TUBB3 781-1179 R | TGCGGATCACTAGTGCTAGCGGCCTTGCGCCGGAACATGGC |
| ShRPPH1-1 F | CCGGACCCAATTCAGACTACTCTCTCGAGAGAGTAGTCTGAATTGGGTTTTTTG |
| ShRPPH1-1 R | AATTCAAAAAACCCAATTCAGACTACTCTCTCGAGAGAGTAGTCTGAATTGGGT |
| ShRPPH1-2 F | CCGGGAGGTGAGTTCCCAGAGAACTCGAGTTCTCTGGGAACTCACCTCTTTTTG |
| ShRPPH1-2 R | AATTCAAAAAGAGGTGAGTTCCCAGAGAACTCGAGTTCTCTGGGAACTCACCTC |
| ShTUBB3-1 F | CCGGGAGCCATTCTGGTGGACCTCTCGAGAGGTCCACCAGAATGGCTCTTTTTG |
| ShTUBB3-1 R | AATTCAAAAAGAGCCATTCTGGTGGACCTCTCGAGAGGTCCACCAGAATGGCTC |
| ShTUBB3-2 F | CCGGGCATGGGCACGTTGCTCATCTCGAGATGAGCAACGTGCCCATGCTTTTTG |
| ShTUBB3-2 R | AATTCAAAAAGCATGGGCACGTTGCTCATCTCGAGATGAGCAACGTGCCCATGC |
